# Estimation of the linear mixed integrated Ornstein–Uhlenbeck model

**DOI:** 10.1080/00949655.2016.1277425

**Published:** 2017-01-12

**Authors:** Rachael A. Hughes, Michael G. Kenward, Jonathan A. C. Sterne, Kate Tilling

**Affiliations:** ^a^ School of Social and Community Medicine, University of Bristol, Bristol, UK; ^b^ Department of Medical Statistics, London School of Hygiene and Tropical Medicine, London, UK

**Keywords:** Fixed effects, Newton Raphson, Integrated Ornstein–Uhlenbeck process, random effects, repeated measures, 62F99, 62J99, 62M10, 62P10

## Abstract

The linear mixed model with an added integrated Ornstein–Uhlenbeck (IOU) process (linear mixed IOU model) allows for serial correlation and estimation of the degree of derivative tracking. It is rarely used, partly due to the lack of available software. We implemented the linear mixed IOU model in Stata and using simulations we assessed the feasibility of fitting the model by restricted maximum likelihood when applied to balanced and unbalanced data. We compared different (1) optimization algorithms, (2) parameterizations of the IOU process, (3) data structures and (4) random-effects structures. Fitting the model was practical and feasible when applied to large and moderately sized balanced datasets (20,000 and 500 observations), and large unbalanced datasets with (non-informative) dropout and intermittent missingness. Analysis of a real dataset showed that the linear mixed IOU model was a better fit to the data than the standard linear mixed model (i.e. independent within-subject errors with constant variance).

## 1. Introduction

Linear mixed models, proposed by Laird and Ware [1], are commonly used for the analysis of longitudinal clinical markers (‘biomarkers’) of disease; for example HIV research on the time course of CD4 counts [2] and time course of progesterone in a menstrual cycle [3]. In such settings the data are typically unbalanced: the number of measurements differs among subjects and the time interval between consecutive measurements differs within and between subjects. The variance of the biomarker may be nonstationary (vary over time) and, when measurements on the same individual are recorded close together in time, within-subject measurements may be serially correlated (also known as autocorrelation). Typically, the linear mixed model is applied assuming no within-subject serial correlation (‘conditional independence’). However, the conditional independence model can allow for nonstationary variance through between-subject random effects: for example, a model with a random intercept and a random linear slope implies that the variance is a quadratic function of time. The stochastic linear mixed model is the conditional independence model with an added Gaussian stochastic process to model within-subject serial correlation. Diggle [4] proposed a stochastic linear mixed model where the covariance structure was assumed to be stationary and modelled by a random intercept, independent measurement errors and a stationary Gaussian stochastic process (the Gaussian correlation function). Taylor et al. [5], Zhang et al. [6] and Stirrup et al. [7] allowed for a nonstationary covariance structure by including in their model random effects other than the random intercept and a nonstationary stochastic process (the integrated Ornstein–Uhlenbeck (IOU) process, the nonhomogeneous Ornstein–Uhlenbeck process and the fractional Brownian Motion process respectively). This paper focuses on the model proposed by Taylor et al., which we shall refer to as the linear mixed IOU model.

Based on model fit statistics (Akaike Information Criterion and log-likelihood) Taylor et al. [5] and Wolbers et al. [8] concluded that, compared to a conditional independence model, the linear mixed IOU model gave an improved fit to longitudinal CD4 counts. Based on a simulation study, Taylor and Law [9] concluded that, when predicting future measurements in subjects, a linear mixed IOU model was more robust than a conditional independence model (with a random linear or quadratic slope) to incorrect specification of the true covariance structure of the data. However the linear mixed IOU model is rarely used in practice, due to the lack of available software.

The purpose of this paper is to describe the estimation and implementation of the linear mixed IOU model within Stata, and to evaluate, using simulations, the feasibility and practicality of estimating the linear mixed IOU model by restricted maximum likelihood (REML) estimation. We compare REML estimation of the model under different: 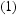 parameterizations of the IOU stochastic process, 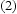 longitudinal data structures, 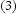 optimization algorithms and 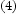 random effects structures. We also evaluate our implementation when applied to unbalanced data typically seen in large cohort studies. In Section 2 we describe the linear mixed IOU model, the estimation procedure and our implementation in Stata 13.1 [10]. Sections 3 and 4 present the methods and results of our simulation study respectively. In Section 5 we apply the linear mixed IOU model to height measurements from Christ's Hospital Study and we conclude with a general discussion in Section 6.

## 2. Computational methods and theory

### 2.1. Linear mixed IOU model

The linear mixed IOU model implies that a subject's biomarker trajectory is a realization of an IOU process. Taylor et al. [5] hypothesized that in the short term a subject's biomarker process may maintain the same trajectory but over long periods of time the biomarker's trajectory may vary. A special feature of the IOU process is that it allows for differing levels of derivative tracking; that is, the degree to which a subject's biomarker measurements maintain the same trajectory over long periods of time. The linear mixed IOU model estimates the degree of derivative tracking from the data as opposed to assuming a fixed level via a particular covariance function. Two limiting cases of the model are 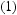 a specific conditional independence model (strong derivative tracking) and 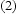 a stochastic linear mixed model with a Brownian Motion process (no derivative tracking).

Consider a dataset of *m* subjects, where subject *i* has 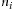 repeated measurements recorded at time-points 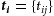 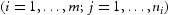. For subject *i*, 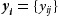 is a 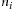-vector of responses, 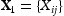 is a 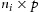 design matrix for the *p*-vector of fixed effects 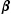 (i.e. population regression coefficients), 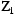 is a 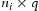 design matrix for the *q*-vector of random effects 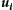 (i.e. subject-specific regression coefficients), 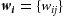 is a 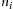-vector of realized values of the IOU stochastic process and 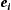 is a 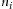-vector of independent measurement errors. The random effects 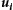, IOU process 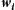 and measurement errors 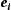 are assumed to be mutually independent. We assume independence of responses 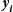 between subjects.

The linear mixed IOU model can be written as

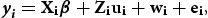
 where 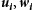 and 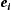 are independently, normally distributed with zero means and covariances 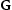, 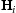 and 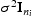, respectively. 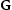 is unstructured and 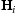 is defined as follows, for 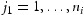, the variance of 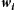 at time-point 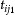 is

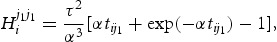
 and, for 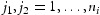 and 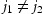, the covariance of 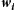 between time-points 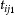 and 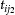 is

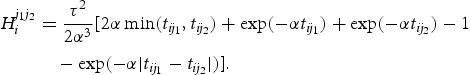



The covariance matrix of 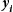 is 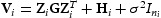 [11] and we denote the vector of unknown variance parameters by 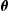, which consists of the unique components of 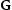, 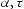 and 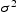. When a linear mixed IOU model only contains a single random effect (a random intercept) we shall refer to the model as a ‘random intercept IOU’ model. Similarly, we shall refer to a linear mixed IOU model with a random intercept and random slope as a ‘random slope IOU’ model.

The IOU stochastic process is parameterized by *α* and *τ*. According to Taylor et al. [5] the *α* parameter can be interpreted as a measure of the degree of derivative tracking, where a small value of *α* indicates a strong degree of derivative tracking (i.e. a subject's measurements closely follows the same trajectory over long periods). The parameter *τ* serves as a scaling parameter.

Taylor et al. [5] note that there are two special limiting cases of the linear mixed IOU model. Firstly, as *α* tends to ∞ (derivative tracking becomes weaker) with 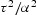 held constant 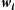 becomes a realization of a Brownian Motion process (also known as the Wiener stochastic process). Taylor and Law [9] called this model the Brownian Motion mixed-effects model. Secondly, for a random intercept IOU model, as *α* tends to 0 (derivative tracking becomes stronger) with 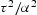 held constant, the model reduces to a conditional independence model with a random intercept and slope, and with zero correlation between the intercept and the slope.

The IOU process is nonstationary because the covariance between 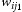 and 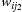 depends upon time-points 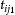 and 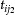 as well as their difference. Due to nonstationarity the covariance matrix of the random intercept IOU model would be the same if it was applied to 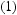 a balanced dataset of measurements recorded at *t* intervals where derivative tracking is *α* and *τ* and 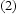 to a dataset of measurements recorded at *Qt* intervals where derivative tracking is 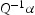 and 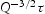. A disadvantage of nonstationarity is that it requires a definition of a natural time 0, which may not be feasible in some applications. In the absence of a known time 0, Taylor et al. [5] proposed modelling differences; for example, selecting a first measurement 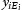 and modelling the differences 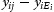 for 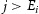. However, this also requires the selection of a ‘first measurement’ which is comparable, with respect to the time course of the disease, for all subjects. Note that, a conditional independence model with random intercept and slope (and an unstructured matrix 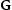) is also nonstationary.

### 2.2. Estimation

Maximum likelihood (ML) estimators of 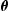 are biased downwards because the estimation process does not take into account the degrees of freedom used in the estimation of the fixed effects 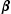 [1,12–14]. For this reason Patterson and Thompson [11] introduced the restricted (or residual) maximum likelihood, a marginal likelihood that is a function of a linearly independent set of residual contrasts and is free of 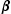. For both REML and ML estimation data can be missing at random [15].

#### 2.2.1. Optimization algorithms

Calculation of REML estimates requires an optimization algorithm such as the Newton Raphson (NR) algorithm [16], Fisher Scoring (FS) algorithm [17], Average Information (AI) algorithm [18] or Expectation Maximization (EM) algorithm [19]. Only the EM algorithm is numerically stable in that the log-likelihood is guaranteed to increase at each iteration and will always converge to estimates within the parameter space. However, the EM algorithm does not give standard errors for the parameter estimates and is slow to converge compared to the NR, FS and AI algorithms [20]. Modified EM algorithms have been proposed to improve the time to convergence; for example using conditional maximization [21–23] and parameter expansion [24, 25].

The FS and AI algorithms are variants of the NR algorithm. The FS algorithm replaces the observed information matrix by the expected information matrix in the NR algorithm, and the AI algorithm replaces the observed information matrix by the average of the observed and expected information matrices (called the AI matrix). The convergence time for the NR algorithm is quicker than the FS algorithm because the NR algorithm converges in fewer iterations and its cost-per-iteration is only slightly slower than that of the FS algorithm [20]. However, the FS algorithm is more robust to poor starting values than the NR algorithm and so Jennrich and Sampson [17] recommended starting with a few iterations of the FS algorithm and then switching to the NR algorithm.

The AI algorithm was developed in the context of animal breeding applications, where estimation can involve manipulation of large matrices. The observed and expected information matrices (used in the NR and FS algorithms respectively) require evaluation of the trace of a matrix. The AI matrix is calculated by taking the sum of squares of expected values, which is quicker to calculate than the trace of a matrix. Gilmour et al. [18] compared the AI and FS algorithms by estimating a linear mixed model when applied to data from a spring wheat trial. The authors concluded that the AI algorithm ‘had similar convergence properties, yet avoided the computing burdens of that approach’ [18].

#### 2.2.2. Implementation of the estimation procedure

Following Pinheiro and Bates [26] we conducted REML estimation with respect to the parameters of the log-Cholesky parameterization of 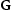. Taylor et al. [5] parameterized the IOU process as *α* and 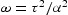 and experienced convergence problems as *α* became increasingly large or small. Taylor et al. suggested (but did not explore) reparameterizing *α* as 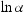 or as 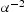 if *α* was suspected to be large. We consider six different parameterizations of the IOU process (see Section 3.1.2, comparison 1): 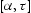, 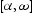, 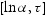, 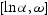, 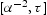 and 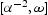.

The covariance matrix of 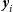 can be expressed as 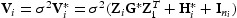, where 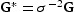 and 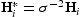. Therefore, we profile the restricted log-likelihood with respect to 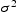 and so reduce the number of parameters to be optimized. We denote the reduced vector of unknown parameters by 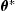, which consists of the unique elements of 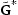 (log-Cholesky parameterization of 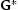), *α* (or 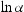 or 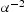) and 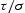 (or 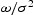). We estimate 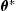 such that it minimizes the negative of twice the profiled restricted log-likelihood for 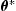. Ignoring the constant terms (and given statistical independence among subjects) the contribution to this function for subject *i* is

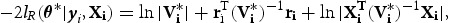
 where 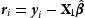 and 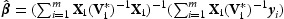 is the estimate of 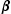 given the latest estimate of 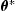.

Once minimization with respect to 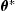 is completed, we transform these parameters such that their ranges are 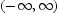. 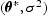 is reparameterized to 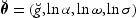, where 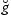 is a vector of the unique elements of 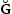, which is a reparameterization of 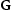 such that the diagonal elements are the logarithms of standard deviations (i.e. 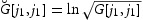) and the off-diagonal elements are hyperbolic arctangents of the correlations (i.e. 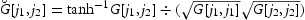 for 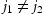). We obtain the information matrix with respect to 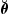, calculate 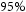 confidence intervals and then transform the endpoints to the required scale (i.e. 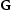, *α*, *ω* and *σ*).

The optimization algorithms require evaluation of the REML log-likelihood and its first and second partial derivatives. When a dataset contains a large number of measurements computation of these quantities can be prohibitively computer-intensive due to the manipulation and inversion of large matrices. As we assume statistical independence between subjects we computed these quantities separately on each subject, which requires the manipulation of (dimensionally) smaller matrices and so improves computational efficiency. To further improve efficiency, we computed these quantities using the method of Wolfinger et al. [27], which avoids direct computation of 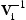 by using an extension of the W-transformation [28,29] and the sweep operator [30].

We programmed the estimation procedure in Stata 13.1 using its matrix programming language Mata [31]. We used the inbuilt Mata *optimize* function to perform the optimization.

The optimization algorithm requires starting values for 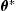. We derived the starting values from the data by calculating, across subjects, variances of the response at measurement time-points and covariances of the response between measurement time-points. For example, for the random intercept IOU model the slope of the variances over time is a reasonable approximation for *ω*. We used the same method for balanced and unbalanced longitudinal data. However, for unbalanced data we first ‘regularized’ the dataset. For example, if measurements were intended to be recorded every 3 months we rounded the ‘observed’ time points to the nearest multiple of 3 months.

## 3. Simulation study design

The aim of the simulation study was to evaluate our Stata implementation of the linear mixed IOU model. The design of the simulation study was based on Taylor et al.'s [5] analysis of the Los Angeles seroconverters cohort of the Multicentre AIDS Cohort Study. The study recruited homosexual and bisexual men in Los Angeles between 1984 and 1985. The cohort consisted of subjects with a known date of seroconversion (i.e. change from HIV antibody negative to HIV antibody positive) and so the natural time zero (for the time course of the HIV biomarkers) was the time of seroconversion. The biomarker of interest was CD4 count (cells/mm^3^) and all CD4 counts were transformed by a fourth-root power to approximate a constant within-subject variance. In the notation of Section 2.1 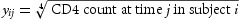. Taylor et al.'s analysis includes all observations before the introduction of AZT treatments. Therefore, CD4 counts tend to decrease with increasing time since seroconversion.

We conducted separate simulation studies based on balanced and unbalanced longitudinal data.

### 3.1. Balanced longitudinal data

The simulation study based on balanced longitudinal data evaluated estimation of the linear mixed IOU parameters under different: 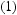 parameterizations of the IOU process, 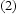 data structures, 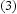 optimization algorithms and 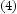 random slope IOU models.

#### 3.1.1. Data model

Consider a dataset with *m* subjects, each with *n* responses (CD4 counts transformed by a fourth-root power) observed at regular *k* monthly intervals (i.e. response 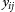 was observed 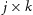 months since time of seroconversion). For subject *i* the *n* responses 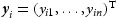 were generated under a linear mixed IOU model with a linear population slope

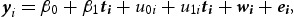
 where 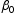 and 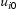 respectively denote the fixed and random intercept, 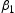 and 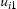 respectively denote the fixed and random slope, and 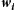 and 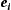 respectively denote realizations of the IOU and measurement error processes. For all settings 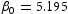, 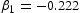 and 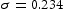, where 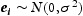. For comparisons 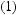 parameterizations of the IOU process, 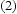 data structures and 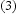 optimization algorithms, data were generated under a random intercept IOU model (i.e. 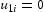 for all subjects) and the variance of the random intercept 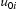 was set to 0.1156. The values of the random effects covariance parameters of the random slope IOU models are discussed in Section 3.1.2 (*Comparison 4*). For all comparisons we repeated the simulations for three levels of derivative tracking: 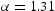 and 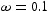 (strong derivative tracking), 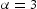 and 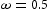 (moderate derivative tracking), and 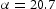 and 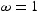 (weak derivative tracking). These values were based on analyses reported in [5, 9].

#### 3.1.2. Comparisons

For all comparisons we generated 1000 datasets and to each simulated dataset we fitted the same linear mixed IOU model that was used to generate the data.

*Comparison* 1: *Parameterization of the IOU process.* We compared six different parameterizations of the IOU process: 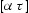, 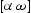, 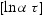, 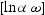, 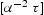 and 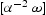. We generated data (under a random intercept IOU model) for *m*=1000 subjects each with *n*=20 measurements recorded every *k*=3 months. The values of *m*,*k* and *n* are typical of large HIV cohort studies. Estimation was performed using the NR algorithm.

*Comparison* 2: *Data structures*: We considered how the estimation process was influenced by: 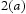 the number of subjects versus the number of measurements per subject (whilst keeping the time interval between consecutive measurements the same), and 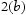 the time interval between consecutive measurements (whilst keeping the number of subjects and the number of measurements per subject constant).


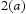
: For a large dataset of 5000 observations in total we compared the following two data structures: *m*=1000 subjects, *n*=5 measurements, *k*=3 months and *m*=250 subjects, *n*=20 measurements, *k*=3 months. Also, for a moderately sized dataset of 500 observations in total we compared data structures: *m*=100 subjects, *n*=5 measurements, *k*=3 months and *m*=25 subjects, *n*=20 measurements, *k*=3 months.


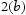
: As discussed at the end of Section 2.1, the degree of derivative tracking depends upon the time interval *k*. By choosing values for *α* and *ω* we were able to change the time interval *k* whilst keeping the degree of derivative tracking the same across comparisons. We compared the following three settings (which result in the same IOU covariance matrix): monthly intervals with 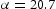 and 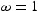, 3-monthly intervals with 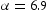 and 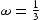 and yearly intervals with 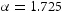 and 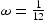. We repeated the simulations for a large dataset of 1000 subjects each with 20 measurements and for a moderately sized dataset of 100 subjects each with 5 measurements.

For both data structure comparisons, estimation was performed using the NR algorithm and the optimal IOU parameterization based on the results from comparison 1.

*Comparison* 3: *Optimization algorithms*: We compared three optimization algorithms: the NR algorithm, a combination of *c* iterations of the AI algorithm followed by the NR algorithm, and a combination of *c* iterations of the FS algorithm followed by the NR algorithm. We repeated the simulations for *c*=3 and *c*=10. We compared the algorithms on two different data structures: a large dataset of *m*=20,000 subjects each with *n*=20 measurements recorded every *k*=3 months and on a moderately sized dataset of *m*=100 subjects each with *n*=5 measurements recorded every *k*=3 months, where starting values (calculated from the data) may be further from the truth for the smaller dataset. Estimation was performed using the optimal IOU parameterization based on the results from comparison 1.

*Comparison* 4: *Random slope IOU models*: We investigated the conditions under which it was feasible to estimate a model with a random intercept, random slope effect and the IOU process. We compared three random slope IOU models with different values for the random effects covariance matrix. Model RS1: 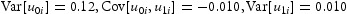 (smallest variances and covariance), model RS2: 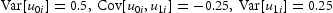 and model RS3: 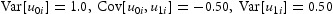 (largest variances and covariance). Estimation was performed using the NR algorithm and the optimal IOU parameterization based on the results from comparison 1.

### 3.2. Unbalanced longitudinal data design

We evaluated our implementation of the linear mixed IOU model when applied to unbalanced data. We allowed the number of measurements per subject and the times of the measurements to vary between subjects. Also, the time intervals between consecutive measurements were allowed to vary between subjects and within a subject. The features of the unbalanced data were based on a UK HIV cohort [32].

#### 3.2.1. Data model

We simulated data on 1489 subjects with 20,000 observations in total: 150 subjects with 2 measurements, 224 subjects with 5 measurements, 372 subjects with 10 measurements and 743 subjects with 20 measurements. We considered two scenarios regarding the time intervals between consecutive measurements.

*Scenario* 1: *Target visits*: All subjects were intended to be measured every 3 months. For subject *i*, the time intervals between consecutive measurements were modelled in days as 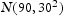 so that 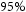 of the time intervals would be between 1 and 5 months duration. All time intervals were required to be at least 7 days.

*Scenario* 2: *Intermittent dropout*: Data were simulated allowing for long gaps between consecutive measurements thus generating data with intermittent dropout. For those subjects with 2 or 5 measurements the time intervals between consecutive measurements were modelled as 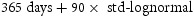, where 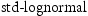 denotes the standardized form of the lognormal distribution 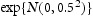. So, 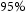 of the time intervals would be between 6 and 18 months long. We simulated under the positively skewed lognormal distribution to avoid negative time intervals. For those subjects with 10 or 20 measurements the time intervals were modelled in days as 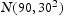. All time intervals were required to be at least 7 days. Under both scenarios, the responses 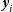 of subject *i* were generated under a random intercept IOU model with a linear population slope using the same parameters values as discussed in Section 3.1.1.

### 3.3. Evaluation criteria

We used the same evaluation criteria for the simulation studies on balanced and unbalanced longitudinal data. We classified the estimation process as converged if the optimization algorithm converged to a solution within 100 iterations and the information matrix with respect to 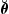 (used to calculate standard errors) was positive definite (i.e. all eigenvalues of the matrix were greater than 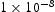). Among the datasets where the estimation process converged we calculated the median and interquartile range (IQR) of the number of iterations to reach convergence. For each variance parameter *ν* we evaluated: (i) bias of the estimates, (ii) empirical standard deviation of the estimates of its transformation 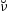, (iii) median and IQR of the estimated standard errors of 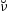 and (iv) coverage percentage of the 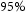 confidence interval for *ν*. Note, we were unable to use the Delta method to approximate the standard errors of 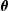 from 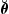 because for some values of 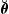 the matrix of first derivatives of the transformation function had zero entries, resulting in zero standard errors. Based on 1000 simulations, the Monte Carlo standard error for the true coverage percentage of 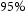 is 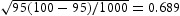 (3 d.p.) [33], implying that the estimated coverage percentage (to nearest whole percent) should lie within the range 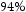 and 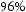 (with 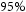 probability).

## 4. Simulation results

### 4.1. Balanced longitudinal data

In this section we present the results for the comparisons of the parameterizations of the IOU process and the random effects structures when derivative tracking was weak (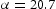 and 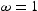). The results for the remaining comparisons (structures of the data and optimization algorithms), and moderate and strong derivative tracking are summarized in the text below. All results not reported are available upon request from the authors.

Across all comparisons, the majority of the fixed effects (linear slope and constant) were unbiased. For those biased estimates the magnitude of the bias was very small; for example, 0.0011 (MCSE 0.0005). Note that, theory tells us that fixed effects REML (and ML) estimators from multivariate normal models are unbiased even when the covariance matrix is misspecified [34]. The confidence intervals for the fixed effects had coverage levels between 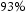 and 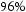.

#### 4.1.1. Parameterizations of the IOU process

Table 1 presents the results of estimation under different parameterizations of the IOU process, where the degree of derivative tracking was weak (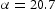 and 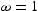). The only IOU parameterization where all estimation processes converged was 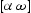. The number of failed estimations due to a non-positive definite information matrix, so that standard errors could not be calculated, was 374,177,13 and 411 for IOU parameterizations 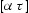, 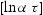, 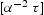 and 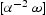 respectively. When examining these failed estimation processes, the estimate for *α* was considerably larger than its true value (e.g. estimate was 51.5 when the truth was 20.7) and the likelihood slice for *α*, with the other parameters held constant at their REML estimates, was flat around the REML estimate of *α*. For parameterizations 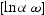 and 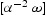 seven estimation processes failed because either the NR algorithm did not reach a solution within 100 iterations or the NR algorithm failed due to a non-positive definite Hessian matrix.

**Table 1.** Comparing parameterizations of the IOU process for balanced data (*m*= 1000, *n*=20 and *k*=3) simulated under the random intercept IOU model with weak derivative tracking 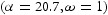.

		Parameterizations					
							
Converged	*N*	626	1000	823	993	987	582
Opt. Iter.	Median (IQR)						
	Bias (MCSE )						
	Empirical SD	0.083	0.096	0.091	0.097	0.093	0.081
	Median SE (IQR)						
	CP						
	Bias (MCSE )						
	Empirical SD	0.55	0.70	0.64	0.73	0.63	0.47
	Median SE (IQR)						
	CP						
	Bias (MCSE )						
	Empirical SD	0.022	0.022	0.022	0.022	0.022	0.022
	Median SE (IQR)						
	CP						
	Bias (MCSE )						
	Empirical SD	0.1	0.13	0.12	0.13	0.12	0.089
	Median SE (IQR)						
	CP						

Optimization performed using NR algorithm. Over 1000 simulated datasets: number converged, median and IQR of the number of optimization iterations (Opt. Iter.) and for each variance parameter *ν*: bias of the estimates of *ν* with Monte Carlo standard error (MCSE), standard deviation of the estimates of its transformation 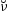, median and IQR of the estimated standard error (SE) of 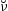 and empirical coverage percentage (CP) of the 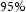 confidence interval for *ν*. Results reported to two significant figures.

Among the estimation processes that converged, the median number of iterations to reach a solution for IOU parameterization 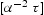 was more than double the median number for the other IOU parameterizations.

Consider the estimation results of the variance parameters for IOU parameterization 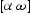. The estimates of the random intercept variance, 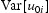, were unbiased and the magnitude of the bias of *ω* was small. Confidence interval coverage for both parameters was close to the nominal level. The distribution of the estimates of *α* and the distribution of its standard errors (of 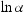) were bimodal. In 469 datasets *α* was overestimated (bias 28.9) and the standard errors of 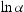 were very large (i.e. median standard error was 14.5 and the standard deviation of the estimates of 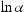 was 0.0972), giving 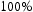 confidence interval coverage. An examination of the likelihood slice for *α* showed a nearly flat likehood around the REML estimate of *α*, which would explain the large standard error. In the remaining 531 datasets *α* was underestimated (bias 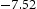) and the standard errors of 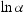 were moderate (i.e. median standard error was 0.352 and the standard deviation of the estimates of 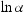 was 0.233), giving 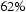 confidence interval coverage. So, over the 1000 datasets there was 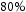 coverage for *α*. For the same reason the confidence intervals for *σ* undercovered. For the other five IOU parameterizations the results of the variance parameters followed similar patterns as noted for 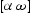.

When derivative tracking was moderate (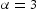, 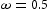) all estimation processes converged for IOU parameterizations involving *τ* and, for IOU parameterizations involving *ω* between 18 (for 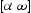) and 28 estimation processes did not converge due to failure of the NR algorithm. When derivative tracking was strong (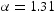, 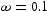) all estimation processes converged for all IOU parameterizations. For strong and moderate derivative tracking the medium number of iterations to reach convergence was between 4 and 7 and the results of the variance parameters were very similar across the 6 IOU parameterizations. The estimates of 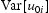, *ω* and *σ* were unbiased, and under moderate derivative tracking *α* was unbiased whereas under strong derivative tracking *α* was only slightly biased (i.e. largest magnitude was 0.0115). For all variance parameters confidence interval coverage was close to the nominal level (95–96 %).

In summary, for weak derivative tracking the preferred IOU parameterization was 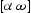, for moderate derivative tracking the preferred IOU parameterization was either 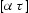, 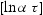 or 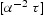, and for strong derivative tracking all IOU parameterizations performed equally well. For the remaining comparisons we estimated the model with respect to the 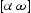 parameterization.

#### 4.1.2. Data structures

*Comparison 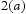: Number of subjects versus number of measurements*: We estimated the model's parameters when applied to two data structures with 5000 observations in total: *m*=1000 subjects each with *n*=5 observations and *m*=250 subjects each with *n*=20 observations. Under weak derivative tracking all estimation processes converged for both data structures. The variance parameter results followed the same patterns as described in Section 4.1.1 for IOU parameterization 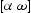 (column 4 of table 1; data structure 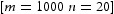). The only notable difference between the data structures was that the confidence interval coverage for 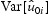 was 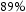 for 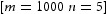 and 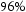 for 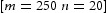. Under strong derivative tracking all models converged for data structure 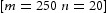, all variance estimates were either unbiased or slightly biased and confidence interval coverage was between 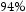 and 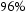. However, for data structure 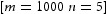 114 estimation processes did not converge within 100 iterations of the NR algorithm. (The same problem occurred, 117 failed estimations, when we used alternative starting values for the optimization algorithm, which were generated assuming strong derivative tracking; that is, the starting values for 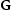 and *σ* were estimated from a standard linear mixed model, and we set 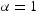 and 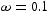.) Among the converged processes the estimates for *α* and *ω* were biased and confidence interval coverage was 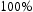 and 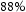 respectively. The estimates for 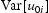 and *σ* were unbiased with close to nominal confidence interval coverage.

We also applied the model to smaller datasets (of 500 observations in total): *m*=100 subjects each with *n*=5 observations and *m*=250 subjects each with *n*=20 observations. Under weak derivative tracking, estimation processes were less likely to converge when the number of subjects was low (4 estimations failed for 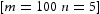 compared to 51 failures for 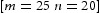). However, under strong derivative tracking estimation processes were less likely to converge when the number of measurements per subject was low (107 estimations failed for 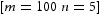 compared to five failures for 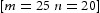). The estimation results of the variance parameters followed the same patterns as noted for the data structures with 5000 observations.

In summary, for datasets of 5000 and 500 observations, the variance parameter results were poorer when the number of measurements per subject was 5 compared to 20 measurements per subject.

*Comparison 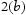: Time intervals between consecutive measurements*: For datasets of 1000 subjects each with 20 observations, we compared estimation of the model when measurements were spaced monthly 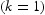, every three months 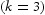 and yearly 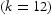. The IOU covariance matrix, 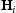, was the same for all three comparisons and equivalent to weak derivative tracking (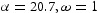 with *k*=1). All estimation processes converged for *k*=1 but 10 and 53 estimations failed for *k*=3 and *k*=12 respectively. The estimates of 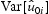 were unbiased for *k*=1,3 and 12. All estimates of *α* were biased and the magnitude of this bias decreased with increasing time interval (i.e. 0.562,0.199 and 0.0546 for *k*=1,3 and *k*=12 respectively). Note that, the true value of *α* was 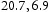 and 1.725 for *k*=1,3 and 12 respectively and, as seen in Section 4.1.1, the magnitude of the bias of *α* was proportional to its true value. Therefore, the magnitude of this bias decreased with increasing time interval because the true value of *α* decreased with increasing time interval. For all time intervals the estimates of *ω* were unbiased and the estimates for *σ* were slightly biased (largest value 0.0045). Confidence interval coverage was close to 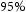 for all parameters and all time intervals.

For moderately sized datasets of 100 subjects each with five observations, all estimation processes converged for *k*=1 but 7 and 1 estimations failed for *k*=3 and *k*=12 respectively. The parameter results followed the same patterns as described for the larger datasets, but the differences in the magnitude of the biases of *α* were larger.

In summary, convergence was most successful when measurements were spaced at monthly intervals. Only the results of parameter *α* differed between the time intervals, where a larger bias occurred for higher values of *α*.

#### 4.1.3. Optimization algorithms

For datasets of 1000 subjects each with 20 observations spaced at 3 monthly intervals, we compared estimation of the model using different optimization algorithms. Under weak derivative tracking, all estimation processes converged when using only the NR algorithm. There were convergence problems with the combination algorithms: 38 estimations failed when the optimizing algorithm was 3 iterations of FS then NR and 60 estimations failed for 3 iterations of AI then NR. Convergence improved when the number of FS and AI iterations preceding NR were increased from 3 to 10, with only 1 failed estimation for 10 iterations of FS then NR and 13 failed estimations for 10 iterations of AI then NR. The median time to convergence was 54 (IQR 89) seconds for NR only, 118 (IQR 93) seconds for 10 iterations of FS then NR, and 136 (IQR 88) seconds for 10 iterations of AI then NR. The results of the parameter estimates of all combination algorithms were almost identical to the results for estimation using only the NR algorithm (column 4 of Table 1). Under strong derivative tracking all estimation processes converged for all optimization algorithms (NR algorithm only and all combination algorithms). The only notable difference between the optimization algorithms was the median time to convergence: 20 (IQR 9) seconds for NR only, 99 (IQR 8) seconds for 10 iterations of FS then NR, and 47 (IQR 4) seconds for 10 iterations of AI then NR.

For moderately sized datasets 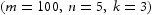 with weak derivative tracking the NR only algorithm had fewer failed estimations: 4 estimations failed for NR only, 10 and 9 estimations failed respectively for 3 and 10 iterations of FS then NR, and 29 and 30 estimations failed respectively for 3 and 10 iterations of AI then NR. The median time to convergence was 1 (IQR 1) second for NR only and 3 (IQR 1) seconds for 10 iterations of FS or AI then NR. When the derivative tracking was strong the algorithm with the least number of failed estimations was 10 iterations of AI then NR: 107 failed estimations for NR only, 85 and 73 failed estimations respectively for 3 and 10 iterations of FS then NR, and 76 and 65 failed estimations respectively for 3 and 10 iterations of AI then NR. Among the 107 datasets where the NR only algorithm failed the median starting value of *α* was 10 (IQR 0), where the true value was 1.31. (The starting values for the other parameters were reasonably close to the truth). When we started the NR algorithm using the alternative starting values (generated assuming very strong derivative tracking) the number of failed estimations for the NR only algorithm decreased to 79. Among the converged estimation processes, the median time to convergence was 2 (IQR 10) seconds for NR only, 4 (IQR 11) seconds for 10 iterations of FS then NR and 3 (IQR 11) seconds for 10 iterations of AI then NR.

In summary, for large datasets with regular and frequent measurements the preferred optimization algorithm was NR only, with respect to convergence and the time taken to reach convergence (on average NR only took at least half the time to reach convergence compared to 10 iterations of FS or AI then NR). In circumstances, such as a moderately sized dataset with a low number of measurements per subject, where starting values are far from the truth the combination of 10 iterations of FS or AI and then NR can improve convergence.

#### 4.1.4. Random slope IOU models

Table 2 presents the results of estimating random slope IOU models when derivative tracking was weak. When the magnitude of the variances and covariance of the random intercept and random slope were small (column RE1 in the table) 122 estimation processes failed to converge: 5 estimations did not converge within 100 NR iterations, 48 NR algorithms failed due to a non-positive definite Hessian matrix, and 69 estimations failed because the information matrix was not positive definite (and so standard errors could not be calculated). For the 122 failed estimations we re-estimated the model using the alternative starting values (generated assuming very strong derivative tracking). Out of these 122 re-estimations 116 converged and the estimated values for the covariance term were very close to zero (median 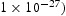. As described by the Stata manual, when an estimated variance component is close to 0 ‘a ridge is formed by an interval of values near 0, which produce the same likelihood and look equally good to the optimizer’ [10]. When this occurs, then the estimation process can fail to converge within a reasonable number of iterations, or fail due to an unstable Hessian matrix, or fail to calculate reliable standard errors [10]. All estimation processes converged when the random effects' variances and covariances were larger (columns RE2 and RE3) and the median number of iterations to reach convergence was almost half the median number required when the random effects were small. For all random slope IOU models, the random effects (co)variance estimates and estimates for *ω* were mostly unbiased (slightly biased in one case) and confidence interval coverage was close to nominal. The results for *α* and *σ* follow the same patterns as noted for the random intercept model when the true value of *α* was large (see Section 4.1.1).

**Table 2.** Comparing random slope IOU model for balanced data (*m*=1000, *n*=20 and *k*=3) simulated with weak derivative tracking 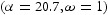.

		Random slope IOU models		
		RE1	RE2	RE3
Converged	*N*	878	1000	1000
Opt. Iter.	Median (IQR)			
	Bias (MCSE )			
	Empirical SD of	0.095	0.036	0.028
	Median SE of (IQR)			
	CP			
	Bias (MCSE )			
	Empirical SD	2.1	0.076	0.053
	Median SE (IQR)			
	CP			
	Bias (MCSE )			
	Empirical SD	0.97	0.043	0.032
	Median SE (IQR)			
	CP			
	Bias (MCSE )			
	Empirical SD	0.68	0.70	0.70
	Median SE (IQR)			
	CP			
	Bias (MCSE )			
	Empirical SD	0.023	0.025	0.025
	Median SE (IQR)			
	CP			
	Bias (MCSE )			
	Empirical SD	0.12	0.13	0.13
	Median SE (IQR)			
	CP			

Note: Optimization performed using NR algorithm and IOU parameterization 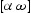. Over 1000 simulated datasets: number converged, median and IQR of the number of optimization iterations (Opt. Iter.) and for each variance parameter *ν*: bias of the estimates of *ν* with Monte Carlo standard error (MCSE), standard deviation of the estimates of its transformation 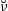, median and IQR of the estimated standard error (SE) of 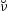 and empirical coverage percentage (CP) of the 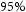 confidence interval for *ν*. Results reported to two significant figures.

When derivative tracking was moderate and strong convergence problems were more common for the random slope IOU model with small random effects variances and covariance (RE1) than for the models with larger random effects' variances and covariance (RE2 and RE3): for moderate derivative tracking 84,25 and 16 estimations failed for RE1, RE2 and RE3 respectively and for strong derivative tracking 10 estimations failed for RE1 and all estimations converged for RE2 and RE3. (Under moderate derivative tracking, convergence improved for all models when we estimated with respect to IOU parameterization 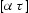. There were more failed estimations for the RE1 model; models RE2 and RE3 had one failed estimation). With respect to the variance parameter results, under moderate derivative tracking, the only notable difference between the random slope IOU models was that the confidence interval coverage for the variance of the random slope term, 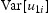, was 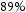 for RE1, and 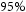 for RE2 and RE3. This confidence interval undercoverage was due to underestimation of the standard errors. Otherwise, under moderate and strong derivative tracking, all parameter estimates were either unbiased or slightly biased (magnitude less than 0.017) and confidence intervals had close to nominal coverage.

In summary, the linear IOU mixed model with a random slope effect can be successfully estimated provided the random effects variances and covariances are not close to zero.

### 4.2. Unbalanced longitudinal data

For unbalanced data generated under the *intermittent dropout* scenario, on average (i.e. taking means over 1000 datasets), the median (IQR), minimum and maximum follow-up time were respectively 4.5 years (IQR 2.4 years), 0.86 and 6.3 years, and the median (IQR), minimum and maximum time interval between consecutive measurements were respectively 3 months (IQR 1.4 months), 1 week and 2.6 years. The number of failed estimations was 78, 28 and 207 for weak, moderate and strong derivative tracking respectively. The estimation processes failed because either the NR algorithm did not reach a solution within 100 iterations or the information matrix was not positive definite. To the same simulated datasets we re-estimated the model using the alternative starting values. For data simulated under strong derivative tracking all 1000 estimation processes then converged. This improvement in convergence occurred because the alternative starting values were closer to the truth than our original starting values. However, for weak and moderate derivative tracking, our original starting values were closer to the truth and so there was no improvement in convergence using these alternative starting values.

Under the *target visit* scenario, on average, the median (IQR), minimum and maximum follow-up time were respectively 3.12 years (IQR 3.29 years), 3.20 months and 6.07 years, and the median (IQR), minimum and maximum time interval between consecutive measurements were respectively 3.00 months (IQR 1.34 months), 1.00 week and 7.01 months. Given the same degree of derivative tracking, the number of failed estimation processes was lower for the *intermittent dropout* scenario than for the *target visits* scenario (106, 118 and 417 failed estimations for the *target visits* scenario under weak, moderate and strong derivative tracking respectively). Successful convergence of the linear mixed model depends upon the number of observations and coverage of the continuous time line of measurements (i.e. from natural time zero to end of follow-up), where an estimation process is more likely to converge the greater the coverage level. Compared to the *target visits* scenario, there was greater variability (between and within subjects) in the times of the measurements for the *intermittent dropout* scenario. Therefore, since data simulated under both scenarios had the same number of measurements, the level of coverage of the continuous time line was higher for the *intermittent dropout* scenario, and hence a greater number of converged estimations.

Among the converged estimations, the median number of iterations to reach convergence (5– 8) and, the estimation results of the fixed effects and variance parameters were very similar between the *target visits* and *intermittent dropout* scenarios. The fixed effects estimates were unbiased and confidence interval coverage was between 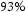 and 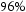 for weak, moderate and strong derivative tracking. Under weak derivative tracking, the estimates of 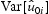 were unbiased and only slightly biased for *ω*, and confidence interval coverage was 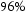. The magnitude of the bias for *α* was at most 1.06 and confidence interval coverage for *α* and *σ* was 92– 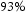. For moderate and strong derivative tracking, all variance estimates were either unbiased or slightly biased (e.g. 0.00313 for *α*) and confidence interval coverage was close to the nominal level.

## 5. Application

We applied the linear mixed IOU model to a large dataset of longitudinal height measurements in 856 boys, aged 9– 18 years, who attended Christ's Hospital School between 1936 and 1969 [35]. The median number of measurements per boy was 60 (IQR 16) and the median time interval between consecutive measurements (within a boy) was 1.3 months (IQR 0.26).

**Figure 1.**
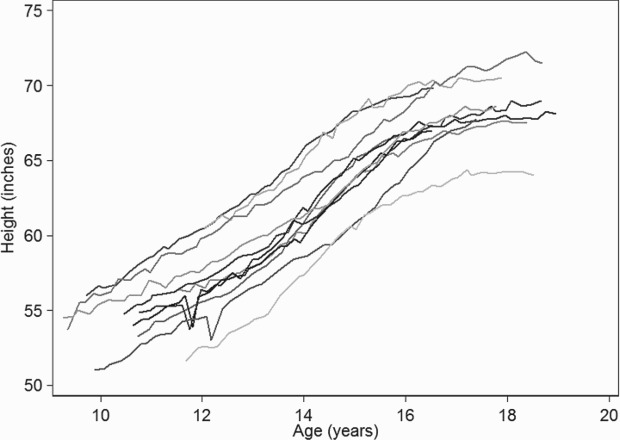
Line plots of height measurements from 10 students who attended Christ's Hospital School between 1936 and 1969.

Figure 1 shows the observed growth trajectories for 10 boys. As the shape of the growth trajectories were close to linear we decided to fit a linear mixed IOU model with a linear population slope.

Lampl et al. [36] state that growth in height in individuals does not necessarily follow a simple trajectory but instead proceeds in a series of jumps separated by period of zero growth. The linear mixed IOU model is ideally suited to allow for variable gradients without the need to specify a complicated function for the growth trajectory. Also, given the frequency of measurements, the linear mixed IOU model allows for any residual serial correlation.

We compared the model fit of a standard linear mixed with a random intercept and random slope, a random intercept IOU model and a random slope IOU model. All models had the same fixed effects and the models with a random slope had an unstructured random effects covariance matrix. The Akaike Information Criterion (AIC) and the Bayesian Information Criterion (BIC) for the linear mixed model, random intercept IOU model and random slope IOU model were respectively AIC=−157,005 BIC=−156,954, AIC=−216,254 BIC=−216,203 and AIC=−216,397 BIC=−216,329. Based on the AIC and BIC values the model with the best fit to the data was the random slope IOU model.

Table 3 presents the results of all three models. The time scale was age in years and the natural time zero was birth. Therefore, the constant fixed effect denotes the average height (in metres) of the boys at birth. The estimates 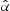 and 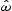 indicate strong derivative tracking. Comparing the results of the standard linear mixed model (RS RE model) and the random slope IOU model we note that the fixed effects estimates differ and the corresponding confidence intervals do not overlap. Given the small magnitude of the estimates for the random effects variances and covariances there was little difference in the fixed effects results and the remaining parameter results between the random intercept IOU model (RI IOU model) and the random slope IOU model.

**Table 3.** Fixed effects and variance parameter results of three models fitted to longitudinal height measurements (in metres) from Christ's Hospital School: the standard linear mixed model with a random intercept and slope (RS RE model), the random intercept IOU model (RI IOU model) and the random slope IOU model (RS IOU model). Results reported to two significant figures.

	RS RE model		RI IOU model		RS IOU model	
	Estimate	Confidence Interval	Estimate	Confidence Interval	Estimate	Confidence Interval
Age (years)	0.055		0.046		0.046	
Constant	0.83		0.94		0.93	
	0.019		0.0073		0.011	
	−0.00087		−	−	−0.00054	
	0.000063		−	−	0.000026	
	−	−	1		1	
	−	−	0.0015		0.0015	
	0.026		0.0086		0.0086	

## 6. Discussion

We have conducted the first evaluation of the feasibility and practicality of REML estimation of the linear mixed IOU model, for both balanced and unbalanced data. Our simulation study demonstrated that the preferred parameterization of the IOU process was 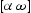 even when the true value of *α* was large. If convergence problems occur with parameterization 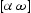 then the user could try parameterization 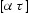, particularly when derivative tracking is neither weak or strong. Estimation of the linear mixed IOU model is feasible for large datasets (i.e. 20,000 observations in total) and moderately sized datasets (i.e. 500 observations). For datasets of 500 observations the model was successfully fitted to data where each subject had 5 measurements each and also to data where the number of subjects was 25. The NR optimization algorithm was preferred over FS and AI algorithms for datasets with regular and frequent measurements. In circumstances, such as a moderately sized dataset with a low number of measurements per subject, where starting values are far from the truth, the combination of 10 iterations of FS or AI and then NR can improve convergence. When applied to a large dataset, the convergence and estimation properties of the random slope IOU model were similar to the random intercept IOU model provided the magnitude of the variance for the random slope effect and its covariance with the random intercept were larger than 0.01. We have demonstrated that it is feasible to apply the model to large unbalanced data with (non-informative) and intermittent dropout.

In our example the linear mixed IOU model was a better fit to the data than the standard linear mixed model and, importantly, inference for the fixed effects coefficients differed between the two models. Taylor et al. [5] showed that the linear mixed IOU model and the Brownian Motion mixed model gave a much better fit to the data (from the Multicenter AIDS Cohort Study) than the standard linear mixed model, but concluded that the choice of the covariance structure made little difference to the point estimates and standard errors of the fixed effects. Using simulations, Taylor and Law [9] compared the linear mixed IOU model, the Brownian Motion mixed model and the standard linear mixed model with respect to the robustness of subject-level predictions when the covariance structure was incorrectly specified. They concluded that the linear mixed IOU model was the preferred model because of its efficiency and robustness properties.

Our method for deriving starting values can only be used when the variance structure is that of the random intercept IOU model or the random slope IOU model. We also considered a general method that can be applied to all linear mixed IOU models, which assumes strong derivative tracking. The rationale behind this method is analogous to the way some commercial software (e.g. SAS, Stata) chooses starting values for a conditional independence model (for example the starting value for 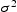 is obtained by fitting a linear regression model to the data using ordinary least squares (OLS) and starting values for the parameters of 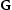 are either 0 or a multiple of the OLS estimate for 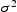). For the linear mixed IOU model we first fitted a conditional independence model (using the EM algorithm). The estimates from this model then form the starting values for the measurement error variance 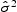 and the random effect parameters of 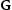. The starting values of *α* and *ω* were set to 1 and 0.1 respectively.

Estimation problems occurred when derivative tracking was weak with large *α*. The most likely cause was a flat likelihood surface near the optimum so that either the optimization algorithm had difficulties converging to a global optimum or it converged with an observed information matrix that was not positive definite, and so standard errors (for the variance parameters) could not be computed. Convergence with valid standard errors given a flat likelihood surface was most likely when the dataset was large and the IOU process was parameterized as 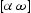. However, estimates of *α* were substantially overestimated with large standard errors (since they were computed from the reciprocal of the curvature of the REML log-likelihood at the optimum). Taylor et al. [5] also reported a very flat likelihood surface for estimates of 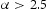 with profile likelihood 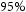 confidence intervals where the upper limit was ∞, with 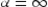 corresponding to one of the special limiting cases of the linear mixed IOU model: the Brownian Motion mixed-effects model. For an estimation process that converges one can diagnose a flat likelihood surface by starting the estimation process with two or more different sets of starting values. If the resulting sets of estimates differ greatly then this indicates a flat likelihood surface near the optimum [37]. The strength of the derivative tracking, as mentioned earlier, depends upon the values of *α* and *ω* and the time intervals between consecutive measurements. For large datasets, these numerical problems did not occur for the scenario of monthly measurements with 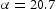 and 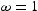; however, these problems were prominent when measurements were every 3 months and derivative tracking was 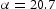 and 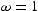, which is equivalent to an IOU covariance matrix based on monthly measurements with 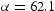 and 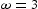.

Convergence problems may be overcome by simplifying the model; for example, removing one or more random effects (apart from the random intercept) from the model, or fitting the Brownian Motion mixed model, which has one less parameter than the linear mixed IOU model, and may be preferable when derivative tracking is suspected to be weak. When convergence problems are suspected to be caused by difficulties in estimating *α*, one suggestion is to derive starting values (of the variance parameters) that are closer to the truth using a computer-intensive approach as follows: first, select values for *α* over an interval that is expected to contain its true value. Second, for each *α* value: fix *α* equal to this value and perform REML estimation with respect to the remaining variance parameters, and record the deviance (−2 x REML log-likelihood). Third, for the model with the lowest deviance use the *α* value and the corresponding estimated parameters as starting values and refit the model where all variance parameters, including *α*, are estimated. This is a simple and, almost, automatic approach that can be easily implemented. However, the approach is likely to be time consuming, especially when searching over many potential values for *α*. Also, setting the interval of *α* to be too narrow and/or too coarse (i.e. large gaps between consecutive *α* values) may omit the true (or close to true) *α* value, thereby generating poor starting values.

Future work could include: 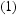 a comparison of the fit and subject-level predictions of the linear mixed IOU model and the Brownian Motion mixed model when derivative tracking is very weak and the likelihood surface of the linear mixed IOU model tends to be flat; 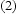 Evaluation of the estimation and fit of the linear mixed IOU model when the population slope is nonlinear (e.g. slope described by a fractional polynomial); and 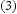 provision of software for the estimation of the Brownian Motion mixed model.

The linear mixed IOU model is a flexible modelling approach but makes parametric assumptions that may not always be well supported by the data. Alternative, non-parametric or semiparametric methods, may be preferred in some situations. For example, the Functional Data Analysis (FDA) approach to longitudinal data provides non-parametric methods, such as smoothing splines, for modelling trajectories [38], where the trajectory (or curve) is the basic unit of data analysis. In general, FDA requires a large number of regularly spaced measurements, such as data on electrical activity of the heart (electrocardiography), electrical activity along the scalp (electroencephalography), and continuous activity monitoring through accelerometers. [39]. However, the development of FDA methodology for longitudinal data, with relatively small numbers of measurements per subject and irregularly spaced, is an area of ongoing research [40], and has been used to model trajectories of prostate specific antigen (a biomarker of prostate cancer) [41, 42]. Also, under certain conditions, the linear mixed model corresponds to a smoothing spline model [43–45], and this connection has been used to develop methods that exploit both features of linear mixed models and FDA [43, 46].

In conclusion, the linear mixed IOU model can provide a better fit to the data than the standard linear mixed model with estimates and confidence intervals for the fixed effects. We recommend estimation of the linear mixed IOU model with respect to the 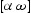 parameterization and using the NR algorithm. If estimation problems occur the user can try our alternative method for deriving starting values and/or estimation with respect to IOU parameterization 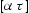. The model can be applied to moderately sized balanced datasets and large balanced and unbalanced datasets. However, estimation problems may occur when the number of measurements per subject is less than 5. One can successfully estimate a linear IOU mixed model with a random slope effect, provided the random effects variances and covariances are not close to zero. When derivative tracking is weak it is important to check for a flat likelihood surface. We have created a Stata command xtmixediou, that fits the linear mixed IOU model, and an accompanying command, predict, to generate predictions under the model. Both of these commands are publicly available from the Statistical Software Components archive.
